# Control of cyclic stability and volume expansion on graphite–SiO_*x*_–C hierarchical structure for Li-ion battery anodes†

**DOI:** 10.1039/d1ra08901c

**Published:** 2022-02-24

**Authors:** Jae Hyeon Yun, Tae Kyung Whang, Won Jun Ahn, Young-Seak Lee, Ji Sun Im

**Affiliations:** C1 Gas & Carbon Convergent Research, Korea Research Institute of Chemical Technology (KRICT) 141 Gajeong-ro Yuseong-Gu Daejeon 34114 Republic of Korea jsim@krict.re.kr; Department of Chemical Engineering and Applied Chemistry, Chungnam University 99 Daehak-ro Yuseong-gu Daejeon 34134 Republic of Korea; Advanced Materials and Chemical Engineering, University of Science and Technology (UST) 217 Gajeong-ro Yuseong-gu Daejeon 34113 Republic of Korea

## Abstract

To increase the energy density of today's batteries, studies on adding Si-based materials to graphite have been widely conducted. However, adding a Si-based material in the slurry mixing step suffers from low distribution due to the self-aggregation property of the Si-based material. Herein, a hierarchical structure is proposed to increase the integrity by using APS to provide a bonding effect between graphite and SiO_*x*_. Additionally, to endow a protection layer, carbon is coated on the surface using the CVD method. The designed structure demonstrates enhanced integrity based on electrochemical performance. The MSG (methane decomposed SiO_*x*_@G) electrode demonstrates a high ICE of 85.6% with 429.8 mA h g^−1^ initial discharge capacity. In addition, the MSG anode has superior capacity retention (89.3%) after 100 cycles, with enhanced volumetric expansion (12.7%) after 50 cycles. We believe that the excellent electrochemical performance of MSG is attributed to increased integrity by using APS (3-aminopropyltrimethoxysilane) with a CVD carbon coating.

## Abstract 2

This study demonstrates the increased integrity of graphite–SiO_*x*_–C hierarchical structure using 3-aminopropyltrimethoxysilane (APS). Furthermore, the carbon coating contributes to elec-trode stability.

## Graphical abstract

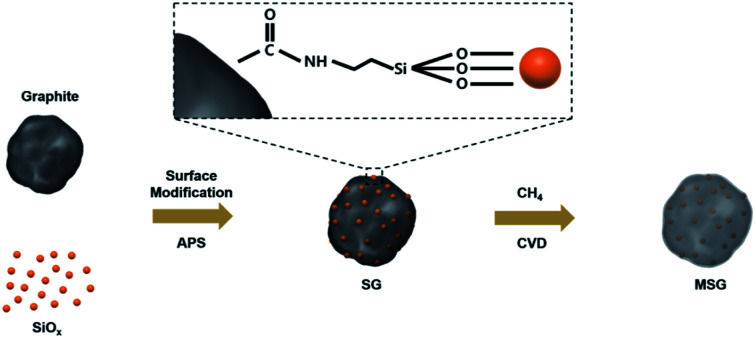

## 1. Introduction

As the electric vehicle market surges, researchers have been striving to increase battery energy density. Because the energy density of lithium-ion batteries (LIBs) is determined by the anode capacity, increasing the anode material's capacity is crucial.^1–4^ For this reason, the introduction of alloy-type materials (Si, Sn, Ge *etc.*) with high energy density to graphite, a commonly used anode material, is being studied.^5–7^ Among these materials, silicon is a major candidate for improving energy density, with a high theoretical capacity of 3572 mA h g^−1^ (Li_15_Si_4_), a low operating voltage (∼0.4 V *vs.* Li/Li^+^), and abundant resources.^4,5^ However, during lithiation/delithiation, silicon suffers large volume changes (∼300%) and has poor electric conductivity.^8,9^ Furthermore, the volumetric expansion leads to atomization of particles, exposing new surfaces, which increases lithium consumption during lithiation/delithiation, and results in a continuous SEI layer with low coulombic efficiency.^6,10^

Compared to silicon, SiO_*x*_ has been studied due to a lower volumetric pulverization (<200%) and relatively high cyclic stability during the lithiation/delithiation process.^11,12^ The lower volume expansion of SiO_*x*_ is caused by products (Li_2_O, Li_4_SiO_4_) that result from the oxygen content of SiO_*x*_ reacting with lithium, which acts as a buffer during the lithiation process.^11,13–15^ However, the generation of Li_2_O and Li_4_SiO_4_, which is an inactive material, consumes a lot of lithium, which leads to a large initial irreversible capacity of SiO_*x*_ anodes.^15–17^ For these reasons, the initial coulombic efficiency (ICE) of SiO_*x*_ anodes is about 70%.^17^

To improve the ICE of SiO_*x*_ anodes, the application of a carbon coating on the surface has been studied. Many methods have been applied to prepare carbon coatings, including mechanical milling,^18^ spray drying,^9,19^ polymer pyrolysis,^20^ and chemical vapour deposition (CVD).^21^ The coating layer of the surface can help build a stable solid electrolyte interface (SEI) layer because of the formation of a uniform surface, as well as improving the electrical conductivity.^22^ The stable SEI layer can increase the ICE of the SiO_*x*_ anodes.^9,23^

In spite of these efforts, the SiO_*x*_ anodes should be blended with commercial graphite anodes due to the low cyclic performance against graphite for industrial applications.^18,24–26^ But, in slurry mixing step, characteristic of the SiO_*x*_ that tends to be aggregated itself interrupts to form uniform slurry. Thus, “integrity” in graphite and SiO_*x*_ anodes must be considered importantly when constructing electrodes.^25,27^ Otherwise, low integrity between graphite and SiO_*x*_ leads to uneven electrode construction, and the composed anode consequently experiences severe volume effects. As a result, the electrode has not only poor electrical contact but also low cyclic stability. To resolve these effects, increasing integrity between graphite and SiO_*x*_ is important for high stability in cycling.

In this article, we developed a hierarchical structure (G–SiO_*x*_–C) to increase the integrity. This type of structure has a main core of graphite and a SiO_*x*_ shell in the subsequent layer and the outermost layer is carbon layer, where SiO_*x*_ is attached to graphite to increase the integrity between graphite and SiO_*x*_. A simple wet-stirring method with 3-aminopropyltrimethoxy-silane (APS) was employed to enhance the integrity of graphite and SiO_*x*_. This could give amino groups on SiO_*x*_, leading to chemical bonding with graphite^28–30^ (hereafter the SiO_*x*_ loaded graphite is denoted as SG). As a result, SiO_*x*_ was homogeneously loaded on graphite. To further increase the integrity of graphite and SiO_*x*_, CVD carbon coating using methane was performed (the methane decomposed SG is hereafter denoted as MSG). The carbon coating of the outermost layers allows the formation of a stable SEI layer and also increases the electrical conductivity. Consequently, we discussed the advantages of this designed structure focusing on (1) improving integrity using APS and (2) surface protection using CVD carbon coating.

## 2. Experimental

### 2.1 Material preparation

#### 2.1.1 Preparation of the SG particle

To prepare SG, we first gave functional groups on graphite and the SiO_*x*_ surface. To functionalize graphite, 4.5 g of artificial graphite (MTI Corporation, D50 = 10.91 μm) was immersed in 20% HNO_3_ solution (20 mL of 60% HNO_3_ and 50 mL of D.I. water mixed solution) and heated at 100 °C for 12 h under stirring. Similarly, to provide functional groups to SiO_*x*_, 0.5 g of SiO_*x*_ (Daejoo Electronic Materials Co Ltd, *D*50 = 660 nm) (Fig. S1†) was immersed in a mixed solution of 10 mL of 98% H_2_SO_4_ and 10 mL of 34.5% H_2_O_2_ and stirred at room temperature for 10 min. This two acid treated solution was rinsed by centrifugation with distilled water several times. The two solutions were then mixed and 120 μL of 3-aminopropyltrimethoxysilane (APS, 95%) was added to the mixed solution and dispersed by sonication. The solution was subsequently stirred for 3 h, and solvent was removed using an evaporator to obtain the SG composite.

#### 2.1.2 Preparation of the MSG particle

To prepare MSG, SG was introduced into a quartz tube furnace. Subsequently, the furnace was heated to 900 °C at a ramping rate of 10 °C min^−1^ under an Ar atmosphere. At the target temperature, a gas mixture of 80 sccm CH_4_ and 20 sccm Ar was flowed. The temperature was maintained for 30 min, and then the furnace was cooled to room temperature in an Ar atmosphere.

### 2.2 Material characterization

The morphology of the prepared samples was evaluated by scanning electron microscopy (SEM, Tescan Mira 3 LMU FEG) with energy dispersive X-ray spectroscopy (EDX, Bruker Quantax 200 XFlash4010) at 10 kV and transmission electron microscopy (TEM, JEM-ARM200F). Structural investigation of the samples was carried out with an X-ray diffractometer (XRD, Rigaku Ultima IV) using Cu Kα radiation (*λ* = 1.5418 Å) and Raman spectra were obtained using a Nanophoton Ramanforce Raman spectrometer with a laser wavelength of 532 nm. The particle size distribution (PSD) of the samples was obtained by a laser particle size distribution analyzer (Microtrac Bluewave). Thermogravimetric analysis (TGA, SDT Q600 V20.9 Build 20) was carried out in air within a temperature range of 25–900 °C. X-ray photoelectron spectrometry (XPS, AXIS SUPRA) was used for measuring the binding energy of the samples with Al Kα radiation. To demonstrate the variation of the electrode after the cycling test, the electrode was washed by dimethyl carbonate in an argon-filled glove box. The electrode before and after cycling was analyzed by scanning electron microscopy – plasma focused ion beam (SEM-PFIB, Helios G4 PFIB CXe DualBeam) with Xe plasma.

### 2.3 Electrochemical measurements

The electrode was prepared by a slurry mixing method on a Cu current collector. The slurry was composed of the active materials (SG, MSG, G/SiO_*x*_*etc.*), conductive (Super P, Imerys Graphite & Carbon), carboxymethyl cellulose (CMC, MTI Corp.), and styrene butadiene rubber (SBR, MTI Corp.) and was uniformly mixed by a Thinky mixer in a mass ratio of 92 : 3 : 2.5 : 2.5 and the loading level of the electrode was 7–8 mg cm^−2^. For the G, the slurry was mixed in a mass ratio of 95 : 2.5 : 2.5 (active : CMC : SBR). The electrode was dried at room temperature for 2 h, and then at 80 °C for 12 h in a vacuum oven and subsequently punched into a circular electrode with a diameter of 13.5 mm. The electrode was calendared for 1.2–1.3 g cc^−1^ of electrode density by a calendaring process. In the cell test, a CR 2032 coin-type cell was used for assembling the cell. The electrolyte was 1.0 M LiPF_6_ in a mixture of ethylene carbonate (EC) : diethyl carbonate (DEC) = 1 : 1 volume ratio and 12 μm of polyethylene (PE) was used as separator. All electrode fabrication was carried out in an argon-filled glove box. The electrochemical properties were measured in a voltage range of 0.01 to 1.5 V *vs.* Li/Li^+^, and all electrochemical tests were estimated with a Wonatech WBCS3000. The cyclic voltammetry (CV) curves were also obtained on a WBCS3000 electrochemical workstation, and the scan rate was 0.1 to 1.0 mV s^−1^ at a voltage range of 0.01 to 1.5 V.

In the full cell test, the cathode electrode was fabricated by the slurry mixing method on a Al current collector with lithium cobalt(iii) oxide (LCO, Alfa Aesar), conductive, and polyvinylidene fluoride (PVdF, Kynar HSV900) in a mass ratio of 94 : 3 : 3 with a solvent of NMP and the loading level of the electrode was ∼17.5 mg cm^−2^. The electrode was dried at 110 °C for 6 h in a vacuum oven, and then cut into a disk with a diameter of 13.5 mm. The electrode was pressed until ∼3.5 g cc^−1^ electrode density. The N/P ratio of the prepared electrode was ∼1.1 and an electrochemical analysis was carried out at a voltage range of 2.7 to 4.3 V. The CR 2032 coin-type cell was used for assembling the full cell. The electrolyte and separator were the same as previously described. All electrochemical tests were performed with a Wonatech WBCS3000.

## 3. Results and discussion

### 3.1 Morphological analysis of hierarchical structure

**Fig. 1:**
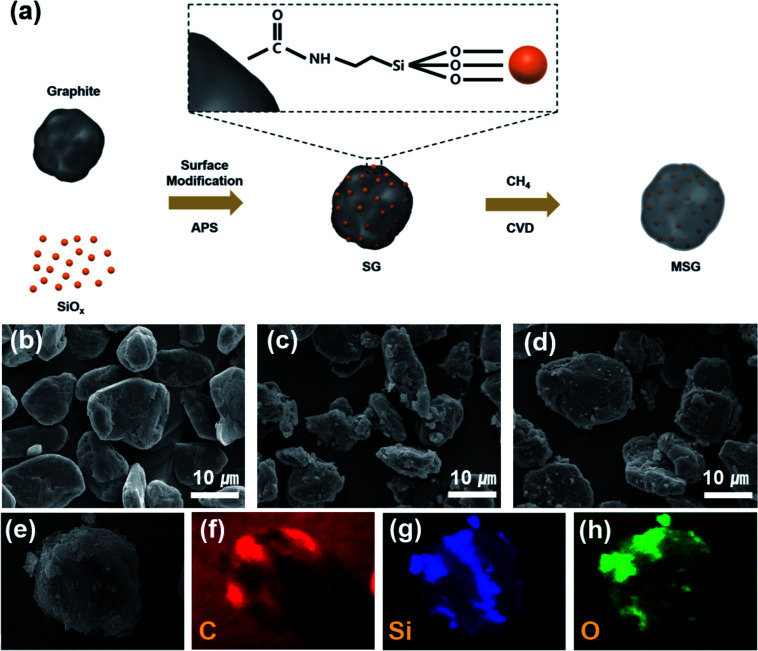
(a) Scheme of the fabrication process of the MSG particles. SEM images of (b) artificial graphite (c) SG, (d) MSG particles. SEM-EDS mapping images of MSG (e–h).

Fig. 1a describes the overall synthesis process of MSG. First, the graphite and SiO_*x*_ were acid-treated to endow carboxylic groups and hydroxyl groups, respectively. Subsequently, the surface treated SiO_*x*_ was stirred in solution to give an amine group by using APS. After that, the SiO_*x*_ was added to graphite and complexed through a condensation reaction between amine and the carboxylic group on the surface of SiO_*x*_ and graphite, respectively. The surface modification of SiO_*x*_ using APS suppresses the tendency of aggregating with each other, allowing it to be loaded regularly into graphite. The MSG was then prepared using the CVD method to form a carbon layer on the SG surface. The carbon coating process was implemented at 900 °C, which was determined by the amount of carbon deposited at different temperatures. When the SiO_*x*_ was reacted at 900 °C, about 5.8 wt% carbon was formed, 13.3 wt% carbon formed at 950 °C, and 24.3 wt% carbon formed at 1000 °C in the TG analysis (Fig. S2†).

**Fig. 2:**
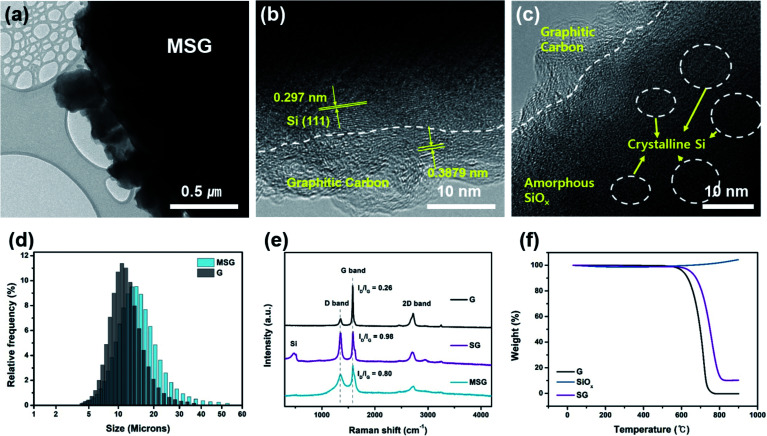
(a–c) TEM images of MSG. (d) Particle size distribution of G, MSG. (e) Raman spectra of G, MSG, SG, SiO_*x*_. (f) Thermogravimetric analysis of G, SiO_*x*_, SG.

To analyze the morphology of the as-prepared samples, SEM was used. As shown in Fig. 1c, in the case of SG, it can be seen that the surface modification of SiO_*x*_ using APS formed a hierarchical structure (G–SiO_*x*_) through a condensation reaction between the amine and carboxyl groups. In Fig. 1d, we can also see that the carbon coated MSG maintains a hierarchical structure (G–SiO_*x*_–C). However, without surface modification, it was found that the SiO_*x*_ was not well placed on the graphite (Fig. S3†). The designed structure was confirmed by SEM-EDS analysis, which showed that the SiO_*x*_ surface was covered by a carbon layer (Fig. 1e–h). The detailed morphology of MSG was elucidated through TEM. In Fig. 2a, as shown in the previous SEM results, we confirmed that the SiO_*x*_ of MSG was well loaded on the graphite surface. From Fig. 2b and c, the deposited carbon coating layer of MSG has about 10 nm thickness and has a graphitic shape with about 0.3879 nm of *d*-spacing, representing the (002) plane. In addition, the SiO_*x*_ showed crystalline Si embedded in an amorphous matrix, and the *d*-spacing of Si is 0.297 nm, representing the (111) plane. The SiO_*x*_ was composed with crystalline silicon and amorphous SiO_*x*_. To investigate the oxygen content of SiO_*x*_, XPS analysis was applied as shown in Fig. S4.† The Si 2p peak of the SiO_*x*_ was deconvoluted based on oxidated state of Si^4+^, Si^3+^, Si^2+^, and Si^1+^, which corresponds SiO_2.0_, SiO_1.5_, SiO_1.0_, and SiO_0.5_. Based on XPS spectra, the oxygen content was calculated by the areal ratio of each oxidated state curve. As a result of estimation, the oxygen content of SiO_*x*_ can be exhibited as 1.14 (Table S1†).^31,32^

To verify the compositional characterization of the designed structure, we further confirmed it by various analysis methods. As shown in Fig. 2d, the particle size of the as-prepared MSG is *D*50 = 13.06 μm, which confirmed that the SiO_*x*_ of MSG was well loaded on graphite without agglomeration considering the size of graphite and SiO_*x*_. However, in the case of samples that did not undergo surface modification, the curves show two peaks at 1.03 μm and 11.13 μm, indicating that surface modification played a major role in forming the composite using APS (Fig. S5†). In the X-ray photoelectron spectroscopy (XPS) analysis, it can be seen that a N 1s peak appears in the SG, whereas in the case of the simple mixed G/SiO_*x*_, the N 1s peak does not appear. This is caused by a condensation reaction using APS (Fig. S6†). To confirm the chemical bonding of obtained samples, FT-IR analysis was conducted. As shown in Fig. S7(a),† it was observed that a lot of functional groups exist in the APS, 3-amino-propyltrimethoxysilane ((CH_3_O)_3_–Si–CH_2_–CH_2_–NH_2_). Most of all, the Si–O peak was exhibited at 1192 cm^−1^ and 1086 cm^−1^ and these peaks were found in SG and MSG (Fig. S7(b)†). However, these peaks were not found in G sample. Therefore, it is noted that the as-prepared samples was well bound by APS.^33^ X-ray diffraction (XRD) reveals the crystallinity of G, SiO_*x*_, SG, and MSG (Fig. S8†). It can be seen that pristine SiO_*x*_ has a wide peak of amorphous SiO_*x*_ around 20°, which is the same as seen in SG and MSG, indicating SiO_*x*_ is added to the graphite. Raman spectra are presented in Fig. 2e to further examine the features of as-obtained samples. As shown in the graph of SG, the peak of SiO_*x*_ appears at around 500 cm^−1^. In the graph of MSG, this peak did not appear because the deposited carbon covered the surface. Moreover, the generated carbon characteristics were identified by comparing two peaks that appear at about 1350 cm^−1^ (D band) and 1600 cm^−1^ (G band), which are typically expressed as *I*_D_/*I*_G_. Compared to graphite, the *I*_D_/*I*_G_ ratio of SG is 0.98, indicating an increase in the D band. This is the result of the increased defects causing by modifying the graphite surface. In the MSG, the *I*_D_/*I*_G_ ratio reduces to 0.8, verifying the deposited carbon is graphitic carbon, which is consistent with the HR-TEM results. To verify the SiO_*x*_ content in SG, a thermogravimetric analysis (TG) was conducted in air (Fig. 1f). The amount of loaded SiO_*x*_ was confirmed as around 10 wt%, considering the increased weight causing by oxidation of SiO_*x*_. The amount of carbon coating was calculated as the amount of carbon on SiO_*x*_ (Fig. S2†). In the case of SiO_*x*_, the amount of carbon coating was approximately 5 wt% of its weight, considering the amount of carbon coating and the *D*50 of MSG, and the thickness of carbon coating was around 11.66 nm, which is consistent with HR-TEM.

### 3.2 Structural benefits of MSG in electrochemical characterization

**Fig. 3:**
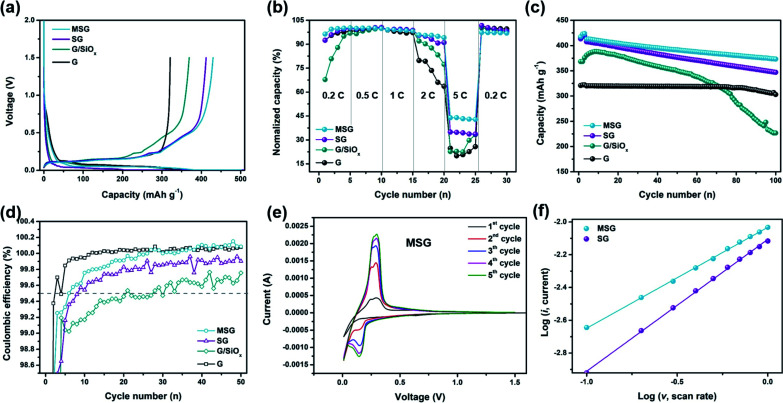
Electrochemical performance in Li-ion half batteries. (a) Galvanostatic charge/discharge voltage profiles of G, G/SiO_*x*_, SG, MSG measured at 0.1C. (b) Rate capabilities of G, G/SiO_*x*_, SG, MSG under increasing *C*-rates from 0.2C to 5C. (c) Discharge capacity of 100 cycles at current density of 1C. (d) Magnified CEs of G, G/SiO_*x*_ SG, MSG for 50 cycles. (e) CV curves of MSG at a scan rate of 0.1 mV s^−1^. (f) log(*i*)/log(*v*) plots of MSG and SG at various scan rates from 0.1 to 1.0 mV s^−1^.

To verify the effect of increased integrity of the MSG electrode, an electrochemical analysis in half cells was tested. As indicated in Fig. 3a, G exhibited a first cycle discharge capacity of 320.9 mA h g^−1^ with an ICE of 91.9%. The first cycle discharge capacity of SG, G/SiO_*x*_ (physically blended in slurry mixing step, G : SiO_*x*_ = 9 : 1 in mass ratio) exhibited 412.2 and 369.2 mA h g^−1^, respectively. The ICE of SG and G/SiO_*x*_ exhibited high performance (82.2% and 80.3% respectively), compared to the ICE of SiO_*x*_ (70%, Fig. S9†). In the case of G/SiO_*x*_ with low integrity, a large amount of Li consumption occurred due to the high reactivity with the electrolyte, leading to a decrease in ICE. In addition, it appears that the capacity was reduced because internal SiO_*x*_ could not participate in the reaction due to self-aggregation between SiO_*x*_. On the other hand, SG increased in both ICE and the capacity, and this is ascribed to the improved integrity of the composite by using APS. Moreover, carbon-coated MSG displayed a higher ICE of 85.6% compared to SG, with an improved discharge capacity of 429.8 mA h g^−1^. This improvement in capacity and ICE originated from the surface carbon coating, which could form a stable SEI layer in the initial charge/discharge process with increased integrity. To increase the discharge capacity of anode material, electrochemical testing of MSG-20 that is prepared with 20 wt% of SiO_*x*_, was conducted. MSG-20 exhibited a first discharge capacity of 526.6 mA h g^−1^, with ICE of 80.6% (Fig. S10†). The slightly lower ICE compared to MSG is due to the small amount of covered carbon layer. The rate performance of the as-prepared samples was measured by varying the discharge rates from 0.2C to 5C (Fig. 3b). In the case of MSG, retention of 5C/0.2C exhibits high capability with an average of 43.4%, compared to 35.3% retention of SG. However, for the simple blended G/SiO_*x*_, retention was very low and similar to that of G (G/SiO_*x*_: 27.0%, G: 23.7%). This also increased the electrical contact between G and SiO_*x*_ using APS, indicating excellent rate capability. In addition, owing to the carbon coating preventing surface exposure between SiO_*x*_ and the electrolyte, MSG exhibits the highest rate capability among the other electrodes. For G/SiO_*x*_, the dispersion was not performed smoothly during the slurry mixing step, resulting in electrochemical activation with increased electrode capacity in the initial five cycles. This was similar in the evaluation of cyclic stability, which was stabilized after about five cycles. The cycle performance was estimated at 0.5C (Fig. 3c). Cycle retention of MSG exhibited high stability, about 89.3% at 100 cycles (84.8% at 150 cycles), which was lower than that of G (94.0%). For the SG and G/SiO_*x*_, cyclic stability was relatively low (SG: 83.8%, G/SiO_*x*_: 67.8%). Notably, this indicated that an increase in integrity also affected cycle performance. Furthermore, the carbon coating through the CVD method covered the SG surface, forming a stable SEI layer that acted to increase the cyclic stability. In Fig. 3d, we describe CE during the cycles. In the case of MSG, the electrode was quickly stabilized and exhibited a high CE of 99.5% after 5 cycles. However, for SG, the electrode reached a CE of 99.5% after 10 cycles, and G/SiO_*x*_ reached a CE of 99.5% after 30 cycles with difficulty. It is expected that this is due to a uniform carbon coating on the surface. In the case of MSG-20, the capacity retention exhibited 83.6% after 50 cycles (Fig. S10†). The lower cyclic stability compared to MSG is due to the increase of SiO_*x*_, implying that the surface carbon layer was insufficient to control the volume expansion of SiO_*x*_.

In Fig. 3e, cyclic voltammetry graphs at a scan rate of 0.1 mV s^−1^ for five cycles are shown. At the first cycle, MSG exhibits a broad cathodic peak at 0.7 V, indicating SEI formation. On the other hand, this peak did not appear in SG, due to the formation of an unstable SEI layer. After the first cycle, the cathodic peak of MSG exhibited two peaks, which corresponded to Li lithiation of graphite (at 0.01 V) and SiO_*x*_ (at 0.15 V, alloying of Li_*x*_Si).^24^ In the anodic peaks at 0.25 V corresponding to the delithiation peak of the graphite,^34^ the delithiation peak of the SiO_*x*_ did not appear. It is expected that the direct reaction of SiO_*x*_ is obscured by the carbon layer on the surface.^24^ For SG, lithiation peaks appear after the first cycle (at 0.01 V of graphite, at 0.15 V of SiO_*x*_), with delilthiation peaks of SiO_*x*_ (at 0.68 V) (Fig. S11†).^35,36^ In both cases of MSG and SG, the peak intensity increased as the cycle proceeded and stabilized after four cycles. Moreover, we investigated the lithium storage kinetics of SG and MSG. A CV test was implemented while keeping the scan rates of lithiation at 0.1 mV s^−1^, with variation of the scan rates of delithiation from 0.1 to 1.0 mV s^−1^ (Fig. S12†). Typically, the peak current and scan rate can be indicated as *i* = *av*^*b*^; by taking logs and plotting log(*i*) *versus* log(*v*), we can determine that the electrode is dominated by diffusion or surface reaction (*b* = 0.5 indicating diffusion-controlled reaction and *b* = 1.0 indicating surface controlled reaction).^37^ As a result of plotting log(*i*) *versus* log(*v*), MSG and SG were 0.61 and 0.67, respectively. This means that the electrode reaction is controlled by diffusion, and a lower value of MSG than SG is a result of graphitic carbon generated on the surface, which is dominantly controlled by diffusion. In other words, this is because graphite is lithiated through Li intercalation while SiO_*x*_ is lithiated through Li alloying.

**Fig. 4:**
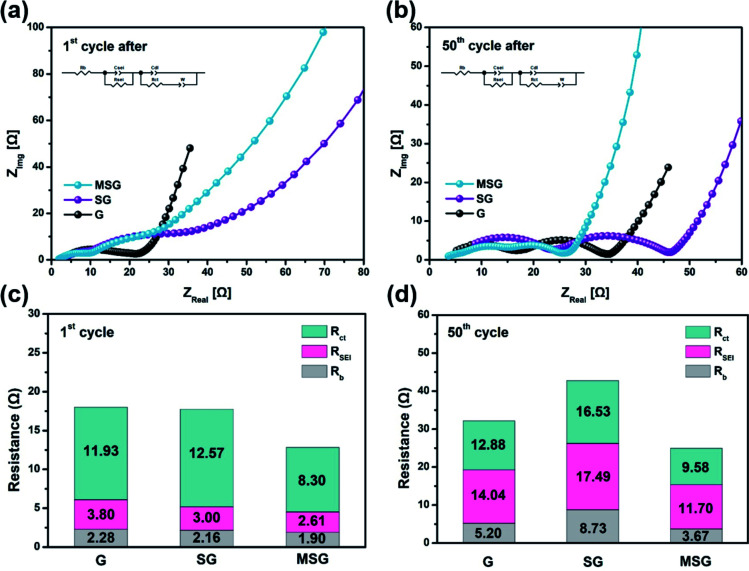
Nyquist plot of (a) G, SG, MSG after 1st cycle and (b) 50th cycles. Charting the Nyquist plot of (c) G, SG, MSG after the 1st cycle and (d) 50th cycles.

To demonstrate the effect on the electrode resistance of the carbon coating, we conducted an EIS analysis. The EIS analysis was implemented in the following circuit, in a completely discharged state after the 1st and 50th cycles (Fig. 4a and b). The *R*_b_, which indicates the electrode bulk resistance, is an index of the State of Health (SOH), representing the sum of the resistance of the electrode, separator, and electrolyte.^38^ The *R*_b_ of G, SG, and MSG increased 2.28, 4.04, and 1.93 times over cycles, respectively. Interestingly, the highest rate of increase was exhibited by SG, which appears to be related to two effects. First, SiO_*x*_ exposed to the electrolyte continues to be atomized with cycling, causing a new SEI; that is, resistance is increased due to electrolyte depletion as the electrolyte is continuously consumed.^39^ Second, the atomized SiO_*x*_ gave rise to a reduced contact area in the internal electrode, which raises the contact resistance, leading to an increase in the electrode resistance. The *R*_SEI_, which indicates the SEI resistance, of the G, SG, and MSG increased 3.69, 5.83, and 4.48 times over cycles, respectively. Likewise in *R*_SEI_, the increased resistance of SG came from excessive SEI formation caused by electrolyte exposure of SiO_*x*_. On the other hand, for MSG, relatively low SEI formation results from low exposure to electrolytes and inhibition of atomization caused by carbon coating. The *R*_ct_, which indicates the electrode charge transfer resistance, showed the highest increase rate in SG. This difference suggests that carbon coating cannot only alleviate volume expansion but also maintains a single structure during repeated cycles, contributing to stable charge transfer. This can be obtained through the Warburg resistance of the straight line, and it can be seen that the diffusion in MSG is higher than in SG (MSG: 3.63 × 10^−9^, SG: 1.04 × 10^−9^ at 1st cycle after) (Table S2†), which means that as the surface is covered with the carbon layer, Li diffusion coefficient increases, and this is consistent with the improvement of the rate performance of the MSG electrodes.

### 3.3 Swelling behavior

**Fig. 5:**
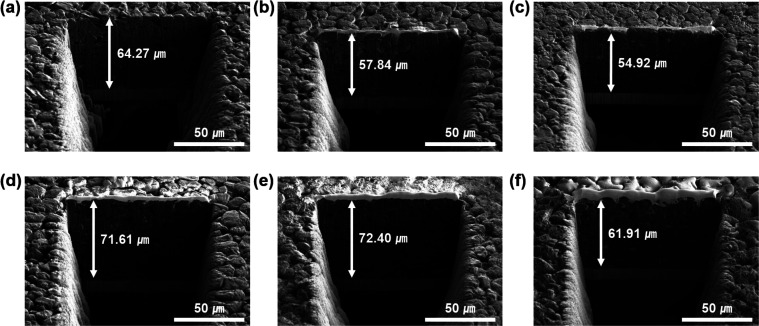
SEM-PFIB images of (a and d) G, (b and e) SG, (c and f) MSG (a–c) before cycling and (d–f) after the 50th cycle, respectively.

To identify the volume deformation of the electrode during lithiation/delithiation, we measured the thickness change of the G, SG, and MSG electrodes using scanning electron microscopy – plasma focused ion beam (SEM-PFIB). In the cycle tests, the electrode was charged at 0.2C rates and discharged at 0.5C rates for 50 cycles. In Fig. 5a and d, G exhibits a volume expansion of 11.4% (71.61 μm after a cycle from 64.27 μm before a cycle), which is similar to the general expansion of graphite in volume of 10%.^40^ However, the electrode of SG exhibited 25.2% swelling (from 57.84 μm to 61.91 μm), which implied the continuous formation of SEI with the surface caused by exposure to the electrolyte. The electrode of MSG, where the surface was covered by a carbon layer, exhibited 12.7% swelling (from 54.92 μm to 61.91 μm), and it increased by only 1.3% compared to that of G. Many trials have been studied to alleviate volume expansion of the electrode such as using porous structure. However, in this experiment, electrode integrity was increased using APS, which leads to alleviate volume expansion.^41–44^ This indicates that volume expansion was successfully alleviated due to the well-designed structure. As shown in the electrode top view before and after the cycles, it can be seen that the degree of damage to the electrode after the cycles is greater than in G and MSG (Fig. S13†). Fig. S14 and S15† present the XPS data of the SG and MSG electrodes after 50 cycles. As shown in the previous electrode expansion results, SG forms an unstable and a thick SEI layer because of electrolyte contact on a new surface. It is confirmed that the Si peak does not appear in the XPS data of the electrode after the cycles (Fig. S14†). On the other hand, in the MSG electrode, the surface carbon layer formed a thin SEI, which was confirmed by a Si peak in the XPS data (Fig. S15†).

### 3.4 Practical application evaluation

**Fig. 6:**
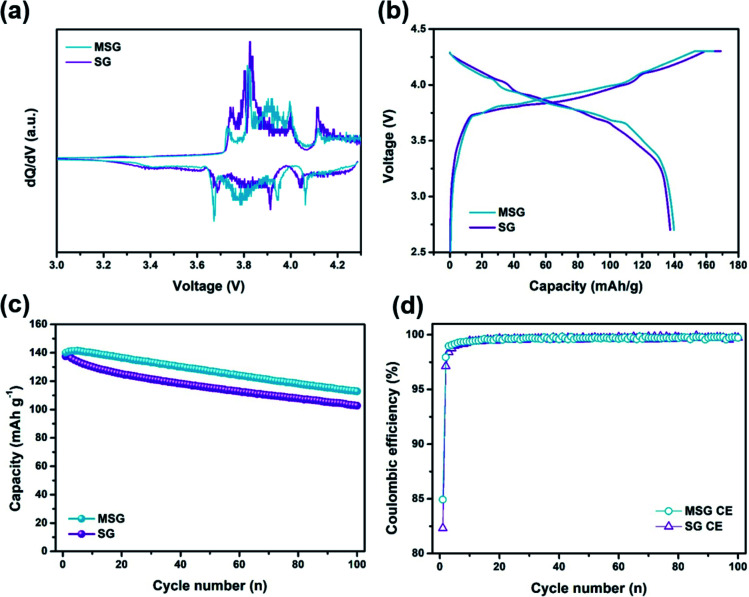
Electrochemical performance in Li-ion full batteries. (a) d*Q*/d*V* plots of SG, MSG at 1st cycle. (b) Galvanostatic charge/discharge voltage profiles of SG, MSG measured at 0.2C. (c) Discharge capacity of 100 cycles and (d) CEs of G, SG, MSG.

With the superior electrochemical performance of MSG, we further investigated practical application of the MSG as an anode in a lithium-ion full fabrication using LiCoO_2_ (LCO) as a cathode. Fig. 6a displays the differential capacity of LCO, indicating general cathodic/anodic peaks of LCO. The capacity of the LCO/SG and LCO/MSG was adjusted to 4.0 mA h with 1.1 of the N/P ratio. The initial discharge capacity of the full cell was 139.9 mA h g^−1^ with the ICE of 84.9% (Fig. 6b). In the case of SG full cell, the initial discharge capacity exhibited 137.4 mA h g^−1^ with an ICE of 82.3%, which is similar to the half cell results. As a result of the cycle test at 0.2C, after 100 cycles, LCO/MSG presented a higher capacity retention of 80.6% than LCO/SG of 74.7%, with stable coulombic efficiency, which was due to stable SEI formation caused by the carbon coating (Fig. 6c and d). It is expected that the increased integrity by using APS can lead to excellent cycling stability. Moreover, carbon coating on the surface can lead to the formation of a stable SEI layer that reduces side reactions caused by the electrolyte with the newly exposed surface. This indicates that the fabricated LCO/MSG electrode can be used in practical applications considering its high performance in cycling.

## 4. Conclusions

In conclusion, we conducted complexation of graphite and SiO_*x*_ through a condensation reaction by surface modification using APS. These new strategies increased the integrity of graphite and SiO_*x*_, which was further increased by carbon coating using the CVD method. This led to a high discharge capacity of MSG (429.8 mA h g^−1^) compared to that of graphite (320.9 mA h g^−1^) and a high ICE of MSG (85.6%) compared to that of SiO_*x*_ (70%). In addition, the MSG electrode exhibits excellent cycling retention of 89% for 100 cycles with the lowest electrode resistance among the measured electrodes, which shows it was well-fabricated in terms of forming a SEI layer. Furthermore, electrode swelling of MSG was 12.7% after 100 cycles, and only increased by 1.3% compared to that of G, indicating controlled volume expansion. Moreover, the fabricated LCO/MSG full cell presents excellent capacity retention of 80.6% for 100 cycles. This is the result of achieving two purposes: (1) preventing electrolyte exposure and (2) maintaining electrical contact by combining graphite and SiO_*x*_ through APS and CVD to form a composite. This attempt fully maintained the high capacity performance of SiO_*x*_ and led to improvement of the cycle performance, which has been vulnerable. Thus, it is considered a strong candidate for introducing a new LIB anode material that exceeds the limitations of existing graphite anodes. We believe that these results are due to the increased integrity of the MSG electrodes. For the commercial use, the ICE of full-cell should be more than 90%. Prelithiaion strategy, to compensate for irreversible Li loss before full-cell fabrication, could be achieve more than 90% of ICE.^45–48^ This is expected to be helpful in practical applications in the future.

## Author contributions

J. Yun: Conceptualization, Investigation, Data curation, Writing – original draft. T. Whang: Investigatioon, Formal analysis, Writing – original draft. W. Ahn: Formal analysis, Investigation. Y. S. Lee: Supervision, Validation. J. Im: Conceptualization, Supervision, Writing – review & editing. All authors contributed to the scientific discussion and the manuscript.

## Conflicts of interest

There are no conflicts to declare.

## Supplementary Material
